# Pattern Visually Evoked Potentials (pVEPs) and Retinal Nerve Fiber Thickness in a Japanese Girl With Anti-myelin Oligodendrocyte Glycoprotein Antibody Seropositive Optic Neuritis

**DOI:** 10.7759/cureus.65254

**Published:** 2024-07-24

**Authors:** Midori Tachibana, Shunichiro Takano, Yuri Ohta, Kei Shinoda, Hideo Yamanouchi

**Affiliations:** 1 Ophthalmology, Saitama Medical University, Iruma-gun, JPN; 2 Pediatrics, Saitama Medical University, Iruma-gun, JPN

**Keywords:** optical coherence tomography (oct), peripapillary retinal nerve fiber (prnfl) thickness, pattern visual-evoked potentials, anti-aqp4 antibody, aquaporin 4 (aqp4), anti-mog antibody, myelin-oligodendrocyte glycoprotein (mog), optic neuritis

## Abstract

We report our findings in a 5-year-old Japanese girl with unilateral optic neuritis who was seropositive for anti-myelin-oligodendrocyte glycoprotein (MOG) antibody. Functional and microstructural changes were assessed longitudinally for 3.5 years by serial recordings of the pattern visual evoked potentials (pVEPs) and optical coherence tomography (OCT) during the acute and chronic phases. On the initial visit, the best-corrected visual acuity (BCVA) in the right eye was light perception. She was treated with 450 mg of intravenous methylprednisolone pulses followed by a gradual tapering of the oral prednisolone. The visual acuity decreased to no light perception, and plasmapheresis combined with high-dose intravenous immunoglobulin therapy was performed. The BCVA quickly improved to 1.0, and no recurrence was detected for approximately four years. The implicit times of N75, P100, and N145 of the pVEPs and peripapillary retinal nerve fiber (pRNFL) thickness in the OCT images were measured during the course of the disease process. The pRNFL thickness of the right eye decreased and was less than one-half of the baseline value at one year and then stabilized. In contrast, the optic pathway function assessed by pVEPs improved. The implicit times of the N75 and P100 components of the right eye were shortened and stabilized at approximately one year. However, the implicit times in the right eye were still longer than that of the left eye. Our findings documented the course of the function and structures of an eye with anti-MOG antibody-positive optic neuritis. This information should be helpful for the understanding of the pathology and prognosis of this disease entity. Further analysis of the pVEPs and structural changes in more cases is needed.

## Introduction

Myelin-oligodendrocyte glycoprotein (MOG) antibody-associated disease (MOGAD) is an identified autoimmune disorder that affects both children and adults as a central nervous system (CNS) demyelinating disorder [1-3]. Clinical presentations are thought to vary by age: children younger than 10 years old are more likely to develop symptoms resembling acute disseminated encephalomyelitis (ADEM), while those aged 10 years and older are more likely to present with a phenotype similar to neuromyelitis optica spectrum disorder (NM SD) or multiple sclerosis (MS) seen in adults [4]. The severity of the attacks is worse, and the recovery is more complete and faster in children [5]. The risk of relapse is lower in children with most having only one incident of the disease [2,6,7].

Analyses of the optical coherence tomographic (OCT) images from MOGAD patients have shown a greater thinning of the papillary retinal nerve fiber layer (pRNFL) in patients with MOGAD than eyes with MS with a history of optic neuritis or NMOSD [8-10]. However, the longitudinal changes in the thickness of the pRNFL in NMOSD-optic neuritis eyes have not been extensively studied [11]. Oetel et al. reported that a recovery from neuroaxonal damage and edema appears to proceed for up to 12 months after optic neuritis, which is longer than what has been reported with other types of optic neuritis [9]. 　　　　　 

Although the visual evoked potentials (VEPs) have proven beneficial for diagnosing and monitoring optic neuritis, only limited information is available on the VEPs in MOGAD patients [3,4,6]. We have reported on the one-year course of the pVEPs in a case of optic neuritis associated with MOGAD [6].

Thus, the purpose of this study was to determine the longitudinal changes in the pVEPs and the thickness of the pRNFL in a young Japanese girl who experienced acute optic neuritis and tested positive for anti-MOG antibodies. The early changes in the pVEPs of this patient have been reported [6]. Serial recordings of the OCT images in addition to the previously reported pVEPs in the acute and chronic phases were made for 3.5 years following the initial diagnosis.

## Case presentation

A 5-year-old girl suddenly experienced a significant decrease in vision along with severe pain in her right eye. The next day, she visited a private eye clinic, where her parents mentioned that she had no history of eye disorders, either personally or within her family. She had no history of viral fever and had not recently received any vaccinations. Her best-corrected visual acuity (BCVA) was measured at 0.7 for the right eye and 1.2 for the left eye. On the second day, a relative afferent pupillary defect was observed in her right eye. Consequently, she was referred to Saitama Medical University Hospital for further evaluation and management.

Our examination found that the BCVA in the right eye was a light perception on day 3. Ophthalmoscopy and OCT revealed swelling of the optic disc and tortuous vessels in the posterior pole of the right eye. No chromatodysopia was detected. The neurological and systemic examinations were within the normal limits. Laboratory tests revealed that all blood parameters were within normal ranges, except for immunoglobulin (Ig)G, which was 680 mg/dl (normal range: 870-1700 mg/dl), and measles IgG-enzyme immunoassay (EIA), which was 18.3 (normal range: 0-1.99 EIA). The cerebrospinal fluid tests were within normal limits except for a pH of 8.0 (normal value, 7.4~7.6). Magnetic resonance imaging (MRI) sequences with short TI inversion recovery (STIR) demonstrated hyperintensities of the right optic nerve. MRI of the spinal cord showed no abnormalities in the cervical, thoracic, and lumbar sections.

She was diagnosed with right papillitis and treated with 450 mg of intravenous methylprednisolone pulses for three days (days 3-5), followed by oral prednisolone with a gradual tapering schedule. On day 5, her visual acuity deteriorated to no light perception, prompting plasmapheresis on days 6, 9, and 12, combined with a high dose of intravenous immunoglobulin therapy (160 mg/kg, total 2.5 g) on day 10. Her decimal visual acuity rapidly improved to 1.0 by day 13. The swelling of the optic disc in the right eye had resolved by day 35, and the retinal vessel tortuosity was no longer present by day 63.

The blood drawn on the initial visit was analyzed on day 9, revealing that the patient was anti-Aquaporin-4 (AQP4) antibody negative. However, she tested positive for anti-MOG antibodies on day 19. The oral prednisolone was gradually tapered off and discontinued after 15 months. Although the patient reported that the image in her right eye appeared darker than in her left eye, her decimal BCVA of the right eye was 1.2 at the last examination at 47 months.

OCT was performed according to the Advised Protocol for OCT Study Terminology and Elements (APOSTEL) and the OSCAR-IB recommendations using the spectral domain OCT Spectralis (Heidelberg Engineering, Heidelberg, Germany; software v. 6.0.12.0). The scans were made with automatic real-time (ART) averaging without pupillary dilation, and they were centered on the optic nerve head (12°, 3.5 mm ring, 50 ≤ ART ≤ 100). The OCT scans were performed several times during the course of the disease process until 47 months after the initial visit [12].

The thickness of the peripapillary retinal nerve fiber layer (pRNFL) was evaluated. pVEPs were recorded multiple times throughout the disease progression and continued after visual recovery. Recording parameters adhered to the standards set by the International Society of Clinical Electrophysiology of Vision (ISCEV), with the exception that the checkerboard size was approximately 2 degrees [13].

**Figure 1:**
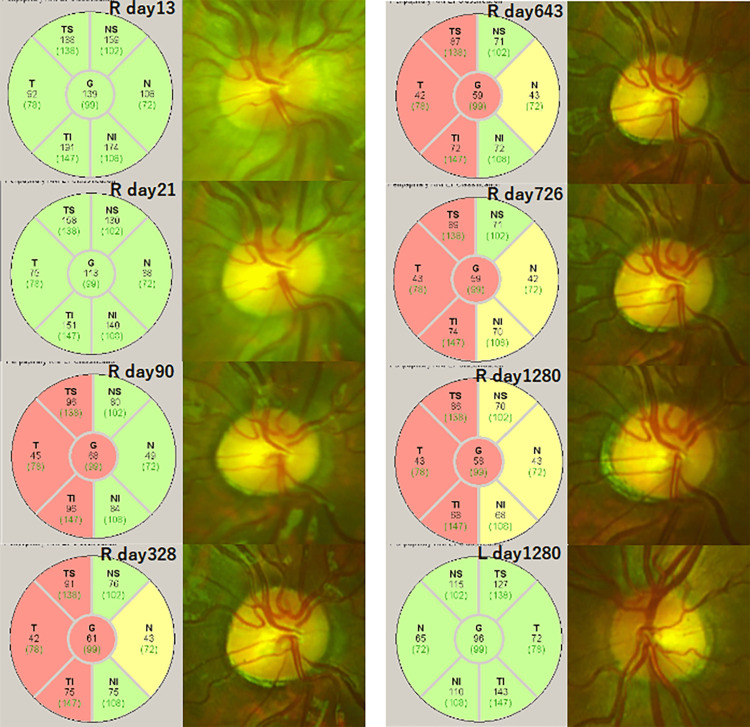
Ring-scan OCT data compared to normative data of the affected and non-affected eyes of the patient Ring-scan optical coherence tomographic (OCT) data compared to normative data of the affected and non-affected eyes of the patient with anti-myelin oligodendrocyte glycoprotein antibody seropositive optic neuritis. The thickness of the peripapillary retinal nerve fiber layer (pRNFL) in the right eye rapidly decreased, becoming thinner than the left eye within 2 months and approximately half within 1 year. Subsequently, it gradually decreased, stabilized around 36 months (day 1092), and remained unchanged for 3.5 years thereafter. The black numbers indicate the thickness measurements (in μm) of the subject, while the green numbers represent the average thickness in the age-matched reference group. Sectors are classified based on comparison with the reference group: green denotes thickness values within the 5th to 95th percentile range, yellow indicates the 1st to 5th percentile range, and red signifies values below the 1st percentile. Abbreviations: G, global; NS, nasal-superior; N, nasal; NI, nasal-inferior; TI, temporal-inferior; T, temporal; TS, temporal-superior.

The pRNFL of the right eye was thicker than that of the left eye during the first 21 days and then decreased to less than that of the left eye on day 34. It then became approximately one-half of the initial thickness on day 62, then gradually decreased and stabilized at approximately 36 months (day 1092). It then remained unchanged for approximately four years (Figure 1).

**Figure 2:**
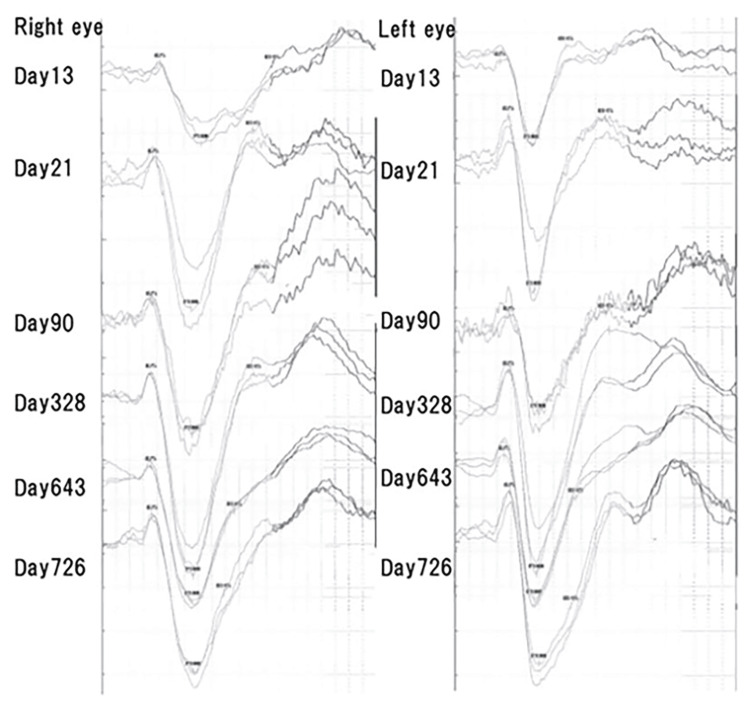
Pattern visual evoked potentials (pVEPs) recorded throughout the course of the disease process. Left Column: pVEPs elicited by stimulating the right eye; Right Column: pVEPs elicited by stimulating the left eye. Top Row: pVEPs recorded on day 13 when visual acuity was 1.0; Second Row: pVEPs recorded on day 21 when decimal BCVA was 1.2; Third Row: pVEPs recorded on day 90 when visual acuity was 1.2; Fourth Row: pVEPs recorded on day 328 when visual acuity was 1.2; Fifth Row: pVEPs recorded on day 643 when visual acuity was 1.2; Bottom Row: pVEPs recorded on day 726 when visual acuity was 1.2. The implicit time of the N75 component was prolonged in the right eye compared to the left eye until day 21, after which it normalized and remained stable for nearly 3.5 years. In contrast, the implicit time of the P100 component remained prolonged in the right eye compared to the left eye throughout the entire follow-up period. The implicit time in the right eye shortened over time and stabilized around four months (day 119), while the implicit time in the left eye remained stable throughout the duration of observation.

The implicit times of the N75 and P100 components of the pVEPs were consistently prolonged when elicited from the right eye compared to the normal left eye throughout the follow-up period (see Figure 2). The implicit times of the right eye gradually shortened until day 119 and remained prolonged up to day 1280 (approximately 3.5 years) in the latest recordings.

**Figure 3:**
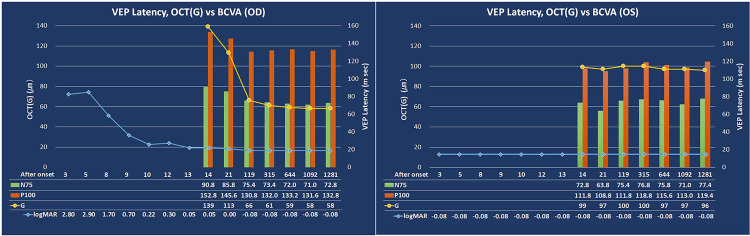
Clinical course of the best-corrected visual acuity (BCVA), implicit times of the pattern visual evoked potentials (pVEPs), peripapillary retinal nerve fiber layer thickness (pRNFL), and treatments A: Right eye. In spite of the thinning of the peripapillary retinal nerve fiber layer thickness (pRNFL) which continued for almost three years, the prolongation of the implicit times of the VEPs stabilized at approximately four months. The BCVA improved rapidly to 0.0 logMAR units by day 13, and there was no recurrence observed for at least 3.5 years. B: Left eye. All parameters remained stable and were within the normal ranges during the course of the disease process. The data are presented as the reference data for the right eye. The green bar shows the implicit time of N75. The red bar shows the implicit time of P100. The orange plot and line show the thickness measurements (in μm) of the global pRNFL. The blue plot and line show the visual acuity in logMAR units. The BCVA of “light perception” and “no light perception” were assigned values of 2.8 and 2.9 logMAR units, respectively.

There was a notable reduction in the pRNFL thickness observed in OCT images, along with prolonged implicit times of the VEPs, which became apparent as the decimal BCVA was nearing complete recovery. These observations reflect changes in the physiological integrity of the visual pathways (see Figures 3A, 3B).

## Discussion

Our study demonstrated longitudinal changes in the implicit times of various components of pVEPs and alterations in the thickness of the pRNFL as observed in OCT images of a 5-year-old girl with anti-MOG antibody-positive optic neuritis. Consistent with earlier reports [5], the attacks were severe, the recovery was fast and complete, lasted for four years, and was monophasic. We found that during the acute phase, the implicit times of the N75 and P100 components were extended in the right eye. Despite maintaining a visual acuity of 1.2 in the right eye for at least one year, the implicit times of the N75 and P100 components of the pVEPs remained prolonged compared to those of the left eye. However, the significance of the correlations between the pVEPs and the thickness of the pRNFL in MOGAD patients has not been determined [6]. Because anti-MOG antibodies seropositive optic neuritis is a relatively new nosological entity, there are only a limited number of studies that investigated the changes in the retinal morphology [4] and the VEPs [3,4] in children. In addition, there are no reports on the longitudinal changes of the pVEPs and thicknesses of the optic nerve during the course of pediatric anti-MOG antibodies seropositive optic neuritis.

In our case, the decimal BCVA initially rapidly improved and then more slowly and reached 1.2 on day 15 after beginning the treatment protocol. The time required for stabilization of the pVEPs and the thicknesses of the pRNFL were clearly different. Both improved several weeks after the improvement of the visual acuity. The pRNFL thinned rapidly for three months and then was stable by 36 months. The implicit times of the pVEP slowly improved after four months and then stabilized, but it was still longer than that of the fellow eye.

The implicit times of the pVEPs were prolonged, and they shortened beginning two weeks after the beginning of the treatment. They then stabilized at four months in the chronic phase but did not return to that of the healthy fellow eye for 3.5 years. Although the long-term recovery of the implicit times of the VEPs is not fully known as in MS eyes with optic neuritis and anti-AQP4 antibody-positive optic neuritis [14], the prolonged implicit times of the pVEPs persisted even after the clinical recovery from the optic neuritis in our case. Vabanesi et al reported that severe axonal atrophy predominated and often blocked identifiable VEP responses in patients with anti-AQP4 antibodies positive NMOSD [14]. Watanabe et al. reported that the P100 was prolonged to more than 121 ms (30′ check-size) in only 1/6 (17%) AQP4+ patients with VEP responses compared to 28/64 (44%) MS patients with prolonged implicit times [15]. They concluded that the lesions were probably more necrotic in the AQP4+ patients and more demyelinating in the AQP4- patients. The severe prolongation of the implicit times in our case may be due to a blocking of neurotransmission by the anti-MOG antibodies seropositive optic neuritis.

The pRNFL of the affected eye was thicker than that of the fellow eye at the onset, then rapidly decreased and became thinner than that of the fellow eye after two months. Then the thickness further decreased and became two-thirds of that of the fellow eye after 11 months. Due to the markedly swollen optic nerve head, which is frequently seen in the acute phase, the pRNFL at the beginning was thicker than that of the fellow eye and then markedly decreased to be less than one-half of the initial thickness. The severe thinning is consistent with earlier reports in eyes with anti-MOG antibodies seropositive optic neuritis [4,16,17]. Despite the severe atrophy of the pRNFL, the BCVA was not altered [16]. Havla et al. reported that patients with pediatric anti-MOG antibodies seropositive optic neuritis had a better visual recovery than adult anti-MOG antibodies seropositive optic neuritis in spite of the profound and almost identical neuroaxonal retinal atrophy [4]. They suggested that age-related cortical neuroplasticity might explain the significant difference observed between the structural changes and the functional outcomes. Although there are no differences in the neuroaxonoretinal atrophy between pediatric and adult MOGAD optic neuritis, it has been argued that neuroplasticity at the cortical level may be the reason why the former had better recovery than the latter [4]. It has been reported that the neurovisual system is not fully mature in children, which supports this neuroplasticity hypothesis [18]. Furthermore, Jenkins et al. reported that the fMRI activity in the lateral occipital cortex is strongly associated with functional outcomes in acute optic neuritis and is a better predictor of visual outcomes in young adults [19].

Costello et al. [20] conducted a prospective study that tracked the RNFL thinning after optic neuritis and reported that the earliest significant interocular differences were manifested two-months after the onset of the optic neuritis and the significant RNFL thinning remained for more than 24 months.

It has been reported that the thickness of the swollen RNFL in eyes with ischemic optic neuropathy decreased to that of normal eyes after one month and was significantly thinner than that of the normal eye after three months [21]. Although our data are from a single case and cannot be generalized, it is notable that our case had characteristics that were similar or had a slightly earlier thinning of the pRNFL thickness compared to them.

## Conclusions

We have presented the longitudinal changes in the implicit times of pVEPs and the thickness of the pRNFL in a 5-year-old Japanese girl with unilateral optic neuritis who tested positive for anti-MOG antibodies over a period of 3.5 years. After a short delay in the visual improvement after beginning treatment, the pVEPs improved but the implicit times remained longer at the latest pVEP recordings at four years. In contrast, even after improvements in the BCVA, the pRNFL thickness became markedly thinner in less than one-half year. The time required for stabilization was clearly different. Assessments of clinical characteristics and analyses of pVEPs and pRNFL thickness are crucial for understanding the pathology of anti-MOG antibody disease. Further case reports and detailed pVEP analyses are expected to offer new insights into diseases associated with anti-MOG antibodies.
